# Lower genital tract cytokine profiles in South African women living with HIV: influence of mucosal sampling

**DOI:** 10.1038/s41598-018-30663-8

**Published:** 2018-08-15

**Authors:** Shameem Z. Jaumdally, Lindi Masson, Heidi E. Jones, Smritee Dabee, Donald R. Hoover, Hoyam Gamieldien, Nontokozo Langwenya, Landon Myer, Catherine S. Todd, Jo-Ann S. Passmore

**Affiliations:** 1 Division of Medical Virology, Institute of Infectious Disease and Molecular Medicine (IDM), University of Cape Town, Observatory, 7925 Cape Town South Africa; 2 Department of Epidemiology and Biostatistics, City University of New York School of Public Health, New York, USA; 3 Department of Statistics and Biostatistics, Rutgers University, Rutgers, New Jersey USA; 4 Centre for AIDS/HIV Program of Research in South Africa (CAPRISA) Centre of Excellence, University of Cape Town, Cape Town, South Africa; 5 Division of Epidemiology & Biostatistics and Centre for Infectious Disease Epidemiology and Research (CIDER), School of Public Health & Family Medicine, University of Cape Town, Cape Town, South Africa; 6 Reproductive, Maternal, Newborn, and Child Health Division, FHI 360 Durham, North Carolina USA; 7 MRC-UCT Gynaecology Cancer Research Centre, University of Cape Town, Cape Town, South Africa; 8 National Health Laboratory Services, Cape Town, South Africa

## Abstract

Measurement of cytokines in the lower female genital tract offer insight into risk for HIV infection and reproductive complications. However, few studies have systematically compared mucosal collection methods or whether collection order matters. We compared longitudinal cytokine profiles in matched genital samples collected from women living with HIV using menstrual cup (MC), endocervical swabs (ECS) and swab-enriched cervicovaginal lavage (eCVL). Samples were collected at enrollment [MC:ECS:eCVL], 3-months (ECS:eCVL:MC) and 6-months (eCVL:MC:ECS) and concentrations of 28 cytokines determined by Luminex. Cytokine clustering was assessed using Principle Component Analysis (PCA), Partial Least Squares Discriminant Analysis (PLSDA) and factor analysis. Generally, higher cytokine concentrations were detected in MC samples, followed by ECS and eCVL, irrespective of study visit or sampling order. Factor analysis and PCA identified ECS to be inferior for measuring regulatory cytokines and IP-10 than eCVL or MC. Although concentrations differed, the majority of cytokines correlated between methods. Sampling order influenced cytokine concentrations marginally, and cytokines clustered more strongly by method than study visit. Variance in profiles was lowest in MC, suggesting greater consistency of sampling compared to other methods. We conclude that MC sampling offered advantages over other methods for detecting cytokines in women, with order marginally influencing profiles.

## Introduction

Accurate measurement of inflammatory cytokine profiles in the female genital tract (FGT) is important to understanding local immune processes that play a crucial role in many infections, diseases and reproductive processes. FGT cytokine concentrations have been associated with HIV acquisition, HIV shedding and transmission, sexually transmitted infections and bacterial vaginosis (BV), adverse pregnancy outcomes, fetal tolerance during pregnancy and cervicovaginal cancers^1–3^. While local cytokines provide protection against viral and bacterial pathogens that commonly invade mucosal surfaces, these cytokines have also been associated with acquisition of some pathogens, like HIV^4,5^. The relative amounts and profiles of cytokines present in genital secretions may vary at different sites in the FGT^6^, possibly reflecting local variations in cytokine-producing cells, innate immune triggers or transient transudation of serum cytokines.

Evaluating factors that cause inflammation in the FGT depends on accurate measurement of immunological biomarkers in genital secretions^4,7^, although few studies have systematically investigated whether anatomical site, method or order of collection influence the composition or concentration of markers detected^8–10^. The FGT mucosa is complex, involving structurally, functionally, immunologically, microbiologically and hormonally distinct zones, and sampling from this region is intrinsically invasive which could influence the biomarkers being measured^11^. The most common approaches to collect secretion samples from the lower FGT include vaginal and endo- or ectocervical swabs (ECS)^12^, cervicovaginal lavages (CVL)^8^ or collection of undiluted mucosal fluid using ophthalmic sponges^13^. Each has been shown to have considerable heterogeneity in their ability to detect and quantify biomarkers. These samples are often dilute (CVLs or diluted cervicovaginal swabs), with a dilution factor that is difficult to determine^14^. CVL is suggested to preferentially capture some immune pathways, further suggesting that sampling approach and site may influence the biomarkers detected^6,15^. There is little consensus on the best approach to mucosal sampling to accurately and reproducibly evaluate immune parameters relevant to prevention or pathogenesis of HIV or other reproductive tract infections (RTIs)^16^.

The menstrual cup (MC) is being widely adopted for mucosal sampling since it is easy to self-insert, comfortably fits over the cervix, and offers the additional advantage of collecting undiluted fluid from the FGT^10,17^. Since MC collects predominantly cervical secretions compared to CVLs and likely causes less mucosal trauma than cervical swabs, a systematic comparison of the MC to these more established mucosal sampling methods before implementing MC widespread introduction in clinical trials with FGT cytokine endpoints is needed. Since some methods of collection are considered to cause more mucosal trauma than others and may influence procedure-induced changes in genital biomarker profiles in subsequent samples, it is important to consider whether the order of genital sample collection matters.

Standardization of collection methods to measure biomarkers at mucosal sites is critical, particularly in prevention trials, as it is important to consider factors that might impact biomarker expression and resistance to pathogens. Here, we compared longitudinal cytokine concentrations and composition in FGT secretions collected using the MC, ECS and swab-enriched CVL (eCVL)^9^ in women living with HIV (WLHIV), and the influence of sampling order on cytokine recovery.

## Results

This study was nested within a blinded randomized controlled trial comparing the levonorgestrel intrauterine device (IUD) and the copper IUD with respect to HIV progression and potential transmission to sexual partners among WLHIV from Cape Town, South Africa (PIs: C.Todd/L.Myer; Trial registration: NCT01721798), during which women were followed for three visits over six months. The 48 women not yet prescribed ART (pre-ART) included in this sub-study had median CD4 counts of 571 cells/mm^3^ (range 357-1,222) at enrollment and plasma viral loads (VLs) of 3.8 Log_10_ copies/mL (range non-detectable [<40] to 5.3 Log_10_ copies/mL). The median genital VL measured in MC secretions was 3.0 Log_10_ copies/mL (range non-detectable to 5.1 Log_10_ copies/mL). We previously reported that genital VLs were significantly higher in MC compared to ECS and eCVL specimens^18^. At the screening visit, 31.3% (15/48) of the women had at least one RTI (including *Neisseria gonorrhoeae*, *Trichomonas vaginalis*, *Chlamydia trachomatis*, and *Treponema pallidum*) or BV diagnosed, all of which were treated prior to enrollment and mucosal sampling.

### Rotation of genital sampling method by study visit

To compare the performance of the MC, ECS and eCVL for collection of genital fluid for cytokine biomarker studies and investigate whether order influenced cytokine concentrations, the sequence of sample collection method was rotated by study visit: Enrollment visit: MC collected first, followed by ECS, and then eCVL; 3-month visit: ECS collected first, then eCVL and finally MC; and 6-month visit: eCVL first, followed by MC, and then ECS.

The median weight of MC specimens collected over the three visits was 0.31 g, ranging from 0.02–1.52 g, which were diluted 1:10 (weight/volume) in PBS. Insertion times for MCs at enrollment (median 114 minutes) were longer than at 3-month (median 70 minutes, p < 0.0001) and 6-month visits (median 73 minutes, p < 0.0001), because they were collected first at the enrollment visit. Insertion time correlated moderately with MC secretion weight (Spearman’s rho = 0.23, 95% confidence interval [CI] 0.06, 0.39; p = 0.006). After adjusting for insertion time, sampling order did not influence MC secretion yield. RTI status at screening did not significantly impact yield of MC secretion (median 0.38 g [range 0.15–1.15 g] for RTI negative versus 0.40 g [range 0.10–1.04 g] for any RTI positive, p = 0.8 at enrollment visit).

### Impact of sampling method on genital cytokine concentrations

**Figure 1:**
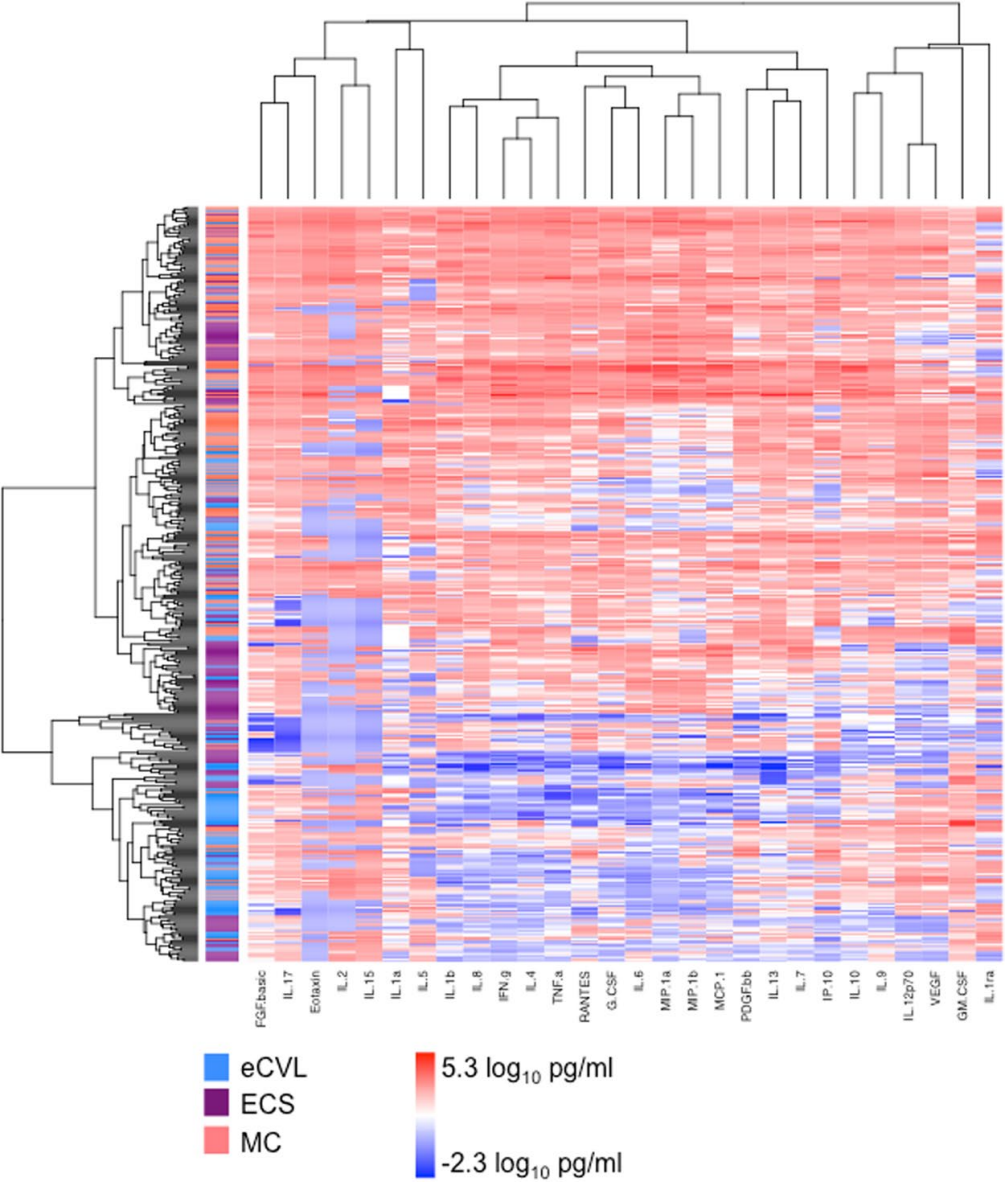
Unsupervised hierarchical clustering was used to visualize the variation in cytokine concentrations in different sample types, irrespective of study visit and cluster the samples according to cytokine concentrations (using the mixOmics package in R). Generally, samples clustered by the method of collection, with cytokine concentrations higher in menstrual cup (MC; coral) samples (n = 142), compared to endocervical swab-enriched cervicovaginal lavage (eCVL; blue) samples (n = 142) and endocervical swab (ECS; dark magenta) samples (n = 142). Cytokine concentrations are indicated using a colour scale, ranging from blue (low) through white to red (high). The dendrogram above the heat map illustrates degrees of relatedness between genital cytokine profiles evident within the various women. The dendrogram on the left hand side of the heat map indicates relationships between the expression profiles of the analysed cytokines across all of the samples assessed in this study.

To compare different methods, the concentrations of 28 cytokines were measured (Fig. 1), including the inflammatory cytokines [Supplementary Fig 1A; interleukin (IL)-1α, IL-1β, IL-6, IL-12p70, and tumour necrosis factor (TNF)-α], chemokines [Supplementary Fig. 1B; IL-8, macrophage inflammatory protein (MIP)-1α, MIP-1β, interferon gamma-induced protein (IP)-10, monocyte chemoattractant protein (MCP)-1, Regulated on Activation, Normal T Expressed and Secreted (RANTES), and eotaxin], adaptive cytokines [Supplementary Fig. 1C; IL-2, IL-4, IL-5, IL-13, IL-15, IL-17, and interferon(IFN)-γ], growth factors [Supplementary Fig. 1D; IL-7, IL-9, granulocyte macrophage-colony stimulating factor (GM-CSF), granulocyte-colony stimulating factor (G-CSF), vascular endothelial growth factor (VEGF), fibroblast growth factor (FGF)-basic, and platelet-derived growth factor (PDGF)-bb], and anti-inflammatory cytokines [Supplementary Fig. 1E; IL-1 receptor antagonist (RA) and IL-10]. The lower limits of detection ranged from 0.01 to 20.97 pg/mL. The median proportion of samples below the limit of detection was 1.9% (ranging from 0 to 53.1%). In paired comparisons, the concentrations of most cytokines were higher in MC than ECS or eCVL, independent of order of sampling (Supplementary Fig. 1), significantly so for IL-1α, IL-12p70, PDGF-bb, and VEGF. Furthermore, the relative concentration (rank) of each cytokine was highly conserved across sample methods. No cytokine was exclusively present or completely absent from any of the three mucosal samples tested.

**Figure 2:**
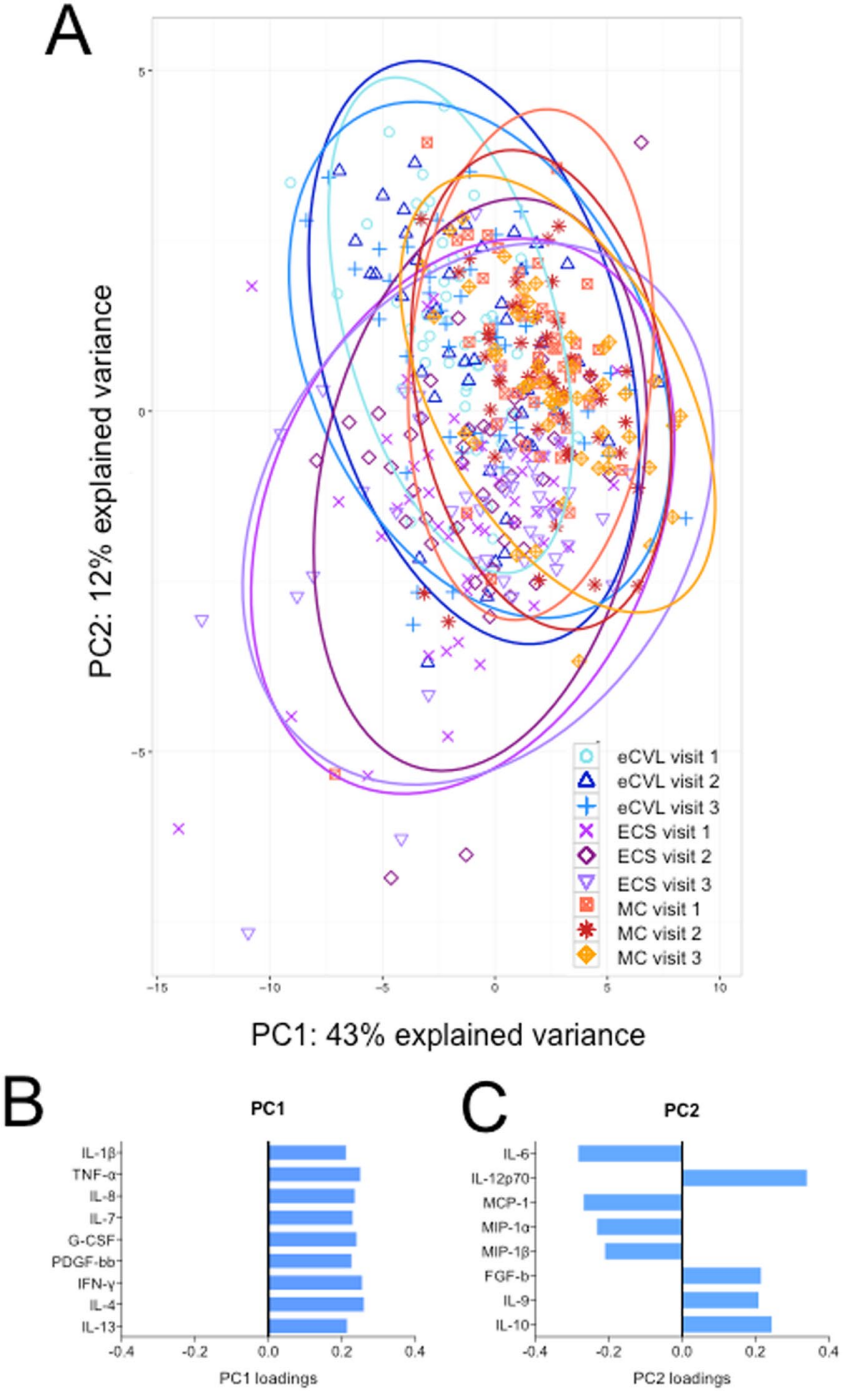
Principle component analysis (PCA) of cytokine profiles by sample type and study visit. (**A**) MC samples are shown in coral (MC visit 1), red (MC visit 2) or yellow (MC visit 3). ECS samples are shown in light purple (ECS visit 1), dark magenta (ECS visit 2) and lilac (ECS visit 3). eCVL samples are shown in turquoise (eCVL visit 1), dark blue (eCVL visit 2), and medium blue (eCVL visit 3). (**B**) The cytokines that loaded positively on PC1 included pro-inflammatory cytokines (IL-1β, TNF-α), chemokines (IL-8), growth factors (IL-7, G-CSF, PDGF-bb) and adaptive cytokines (IFN-γ, IL-4, IL-13). (**C**) The cytokines that loaded positively on PC2 included pro-inflammatory IL-12p70, growth factor FGF-b, and anti-inflammatory IL-10. Cytokines that were negatively associated with PC2 included several chemokines (MCP-1 MIP-1α, MIP-1β) and growth factor IL-9.

**Figure 3:**
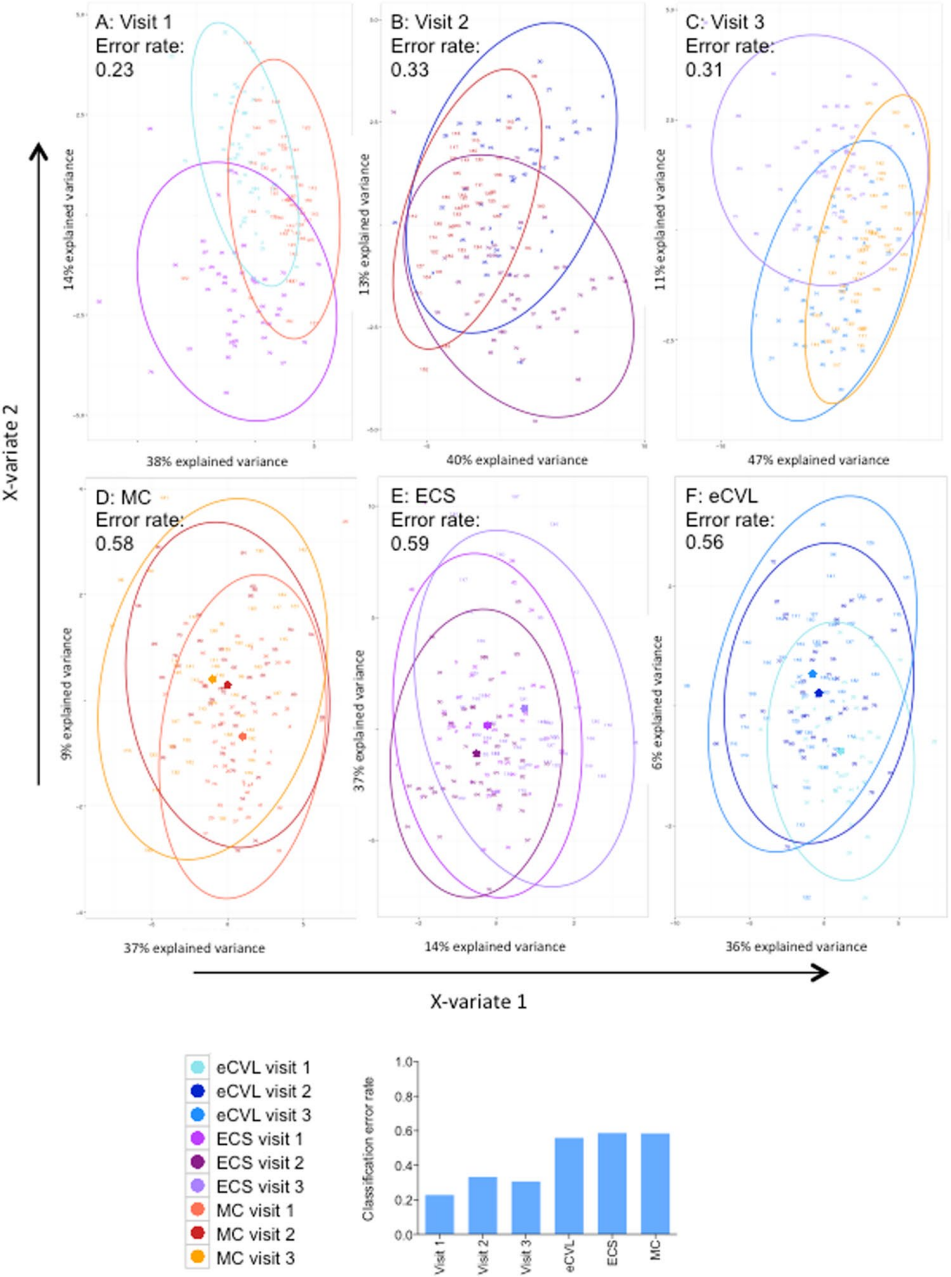
Partial least squares discriminant analysis (PLSDA) was used to cluster samples by cytokine profiles (using the mixOmics package in R). Clustering of different sample types at (**A**) visit 1, (**B**) visit 2 and (**C**) visit 3 and of the same sample types, (**D**) menstrual cups (MC), (**E**) endocervical swabs (ECS), and (**F**) endocervical swab-enriched cervicovaginal lavages (eCVL), at different visits is shown. Cross-validation error rates for all PLSDA models were generated by partitioning the datasets into ten subsets, training the model on nine of these subsets and testing it on one, which was then repeated 50-times for each model. The same sample types at different visits (**D**–**F**) were classified with reduced accuracy compared to different sample types at the same visits (**A**–**C**).

By unsupervised hierarchical clustering of cytokines collected by different methods, genital samples clustered more closely by method than study visit, with MCs consistently having the highest cytokine concentrations, followed by ECS and then eCVL (Fig. 1). Further, unsupervised Principle Component Analysis (PCA; Fig. 2) and supervised Partial Least Squares Discriminant Analysis (PLSDA; Fig. 3) confirmed that samples clustered more strongly by method than study visit. Cytokines associated with PC1 in the PCA included pro-inflammatory cytokines (IL-1β, TNF-α), chemokines (IL-8), growth factors (IL-7, G-CSF, PDGF-bb) and adaptive cytokines (IFN-γ, IL-4, IL-13; Fig. 2B). Cytokines associated with PC2 included pro-inflammatory (IL-6, IL-12p70), chemokines (MCP-1, MIP-1α, MIP-1β), growth factors (IL-9) and anti-inflammatory IL-10 (Fig. 2C). MC samples had higher scores on PC1 than ECS and eCVL, indicating higher concentrations of the cytokines associated with PC1 in MCs versus ECS and eCVL samples. On the other hand, eCVL samples had higher scores on PC2 than MC, followed by ECS. Between 67–77% (cross-validation error: 23–33%) of women were correctly classified when comparing different sample methods at each visit (Fig. 3A–C), whereas only 41–44% (cross-validation error: 56–59%) of women were correctly classified when comparing the same sample methods across multiple visits (Fig. 3D–F). This indicates that cytokine profiles of the same method at different visits were more similar than profiles from different sample methods at the same visit. MC samples also clustered more tightly, with fewer outliers than ECS and eCVL samples (Figs 2 and 3).

**Figure 4:**
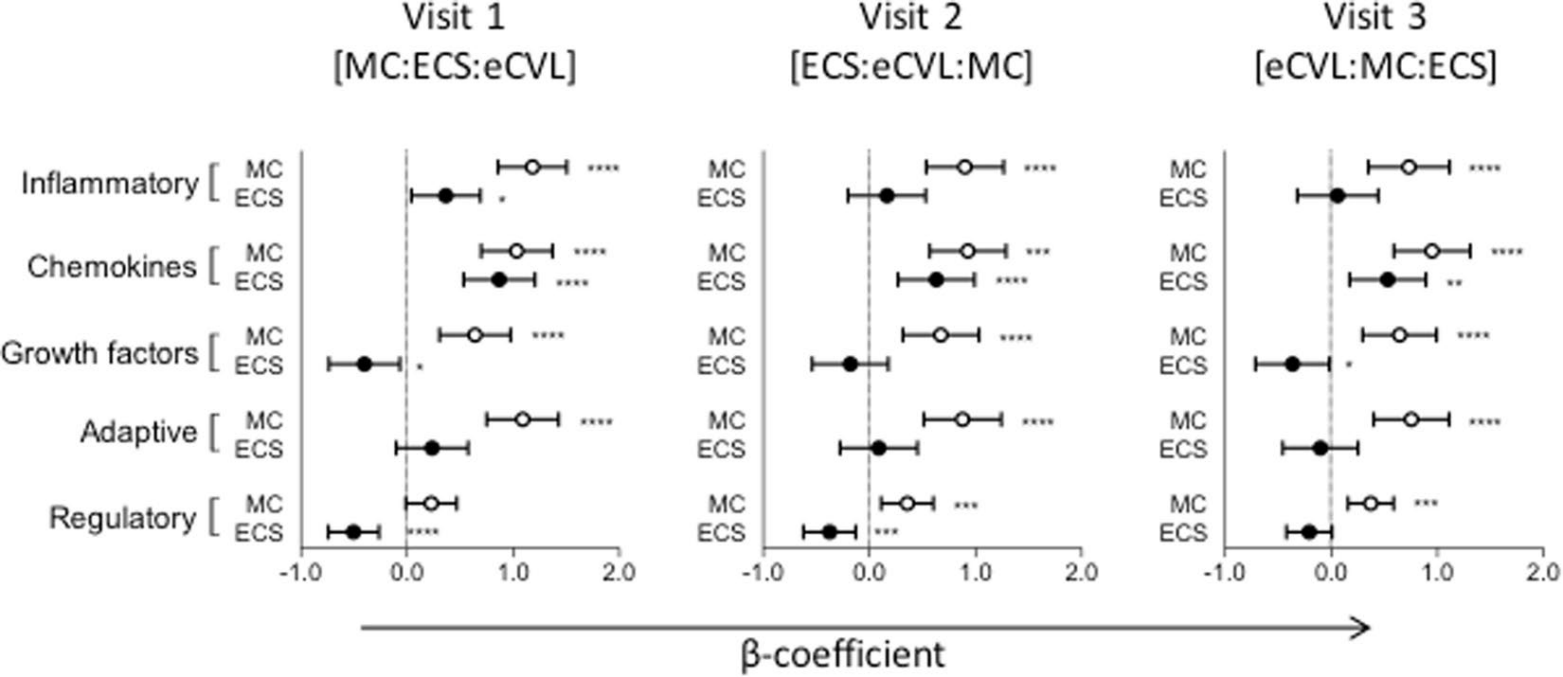
Confirmatory factor analysis of cytokines by functional class. The β-coefficients are shown by the black and open circles for ECS and MC respectively. Cytokine concentrations in eCVL were used as the reference group for statistical models. The error bars represent the 95% confidence intervals. Statistical significance after adjusting for multiple comparisons is shown by asterisks: ^*^p ≤ 0.05; ^**^p ≤ 0.01; ^***^p ≤ 0.001 and ^****^p ≤ 0.0001.

Confirmatory factor analysis was used to simplify the dataset by grouping cytokines into five functional classes/factors (inflammatory, adaptive and anti-inflammatory cytokines; chemokines; growth factors). Factor analysis is useful to account for variability among highly correlated variables like cytokines, to reflect a smaller number of underlying or latent variables. MC and ECS were compared to eCVL (which we considered the gold standard for the purposes of this analysis) (Fig. 4). Compared to eCVL, significantly higher concentrations of all functional classes of cytokines were measured by MC. ECS performed better than eCVL only for inflammatory and chemokine factors, offered little benefit for the growth factors or adaptive cytokines and performed worse than eCVL for regulatory cytokines, irrespective of sampling order or visit number.

**Figure 5:**
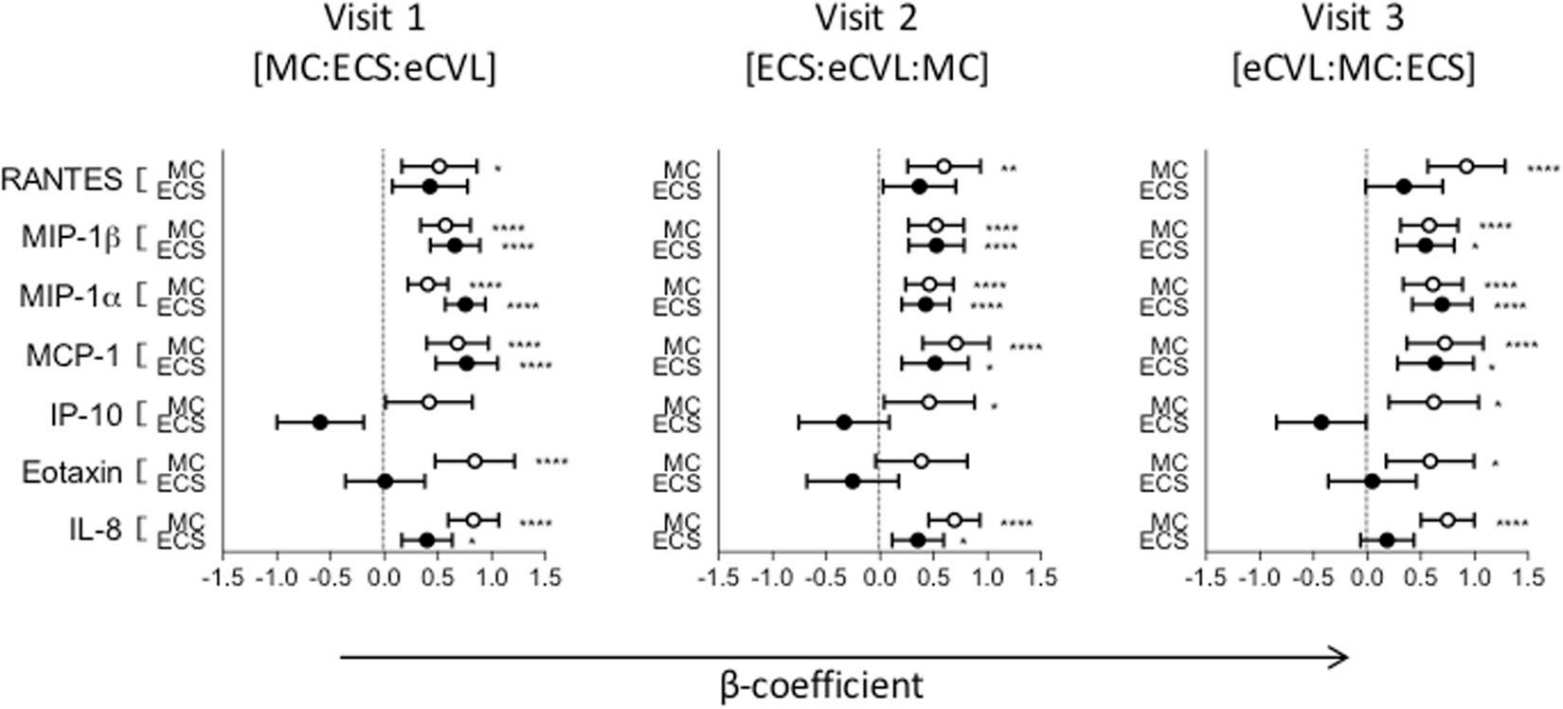
Logistic regression analysis of each chemokine detected in MC (open circles) and ECS (black circles) compared to eCVL as the standard. Regressions were carried out, using cytokine concentrations from eCVL as the reference group. The error bars represent the 95% confidence intervals. Statistical significance after adjusting for multiple comparisons is shown by asterisks: ^*^p ≤ 0.05; ^**^p ≤ 0.01; ^***^p ≤ 0.001 and ^****^p ≤ 0.0001.

Several chemokines (MIP-1β, RANTES, MCP-1), although measured at concentrations well within the range of detection of the assay across sample methods, correlated poorly between MC and ECS, irrespective of sampling order (Supplementary Table 1). Furthermore, some chemokines (MCP-1, MIP-1α and MIP-1β) were detected at slightly higher concentrations in ECS than MC, significantly so for MIP-1α (Supplementary Fig. 1B). In contrast, IP-10 was measured at significantly lower concentrations in ECS compared to either eCVL or MC (Fig. 5 and Supplementary Fig. 1B).

### Correlation between cytokine concentrations measured in distinct sample methods

Although the samples clustered more closely by method than study visit, a large degree of overlap in cytokine profiles between different sample methods was observed by both PCA and PSLDA (Figs 2 and 3). Similarly, the majority of cytokines correlated in matching sample sets, with 26/28 cytokines correlating significantly when MC and eCVL were compared and 24/28 cytokines correlating significantly between MC and ECS (Supplementary Table 1). Concordance between ECS and eCVL cytokine concentrations were most variable, with only 18/28 cytokines correlating. Cytokines that did not correlate between methods tended to be those detected at the lowest concentrations in one or more samples and were least reliably measured across Luminex plates (CV < 80%; such as IL-2 and IL-15; Supplementary Fig. 1C and Supplementary Table 2) or belonging to the chemokine family (MIP-1β, RANTES, MCP-1, Supplementary Fig. 1B).

### Influence of MC insertion time on cytokine concentrations

At the enrolment visit, when MC insertion times were longer and MCs were inserted first, half of the cytokines correlated with MC weight (Table 1). In contrast, at the later study visits, when MCs were inserted second or third and had shorter insertion times, few associations were noted.

**Table 1:** Correlation^*^ between MC weight and cytokine concentrations at each visit.

Class	Cytokine	Visit 1 Median MC weight 0.38 g		Visit 2 Median MC weight 0.29 g		Visit 3 Median MC weight 0.28 g	
		Rho	P-value	Rho	P-value	Rho	P-value
Regulatory	IL-1ra	0,09	0,5934	−0,01	0,9368	0,23	0,1822
	IL-10	0,21	0,1974	0,17	0,3038	−0,08	0,6468
Adaptive	IL-2	0,15	0,3459	0,35	0,0284	0,02	0,8877
	IL-4	**0,49**	**0,0012**	0,3	0,0589	0,07	0,6772
	IL-5	0,10	0,5292	−0,04	0,7984	−0,08	0,6347
	IL-13	−0,06	0,7336	0,17	0,303	−0,12	0,4793
	IL-15	−0,13	0,4129	0,16	0,3141	0,002	0,9898
	IL-17	**0,60**	**<0.0001**	0,16	0,3122	0,12	0,5001
	IFN-γ	**0,41**	**0,0078**	0,26	0,1122	0,11	0,5288
Growth	IL-7	**0,43**	**0,0051**	0,17	0,3087	0,14	0,4324
Factors	IL-9	0,25	0,1108	−0,04	0,8222	−0,12	0,4807
	FGF basic	**0,55**	**0,0002**	0,08	0,6211	0,06	0,7254
	G-CSF	**0,74**	**<0.0001**	**0,51**	**0,0007**	0,29	0,0851
	GM-CSF	0,02	0,9139	−0,13	0,4347	0,001	0,9952
	PDGF-bb	0,17	0,2861	−0,06	0,7176	−0,04	0,8184
	VEGF	0,00	0,9905	−0,19	0,2303	−0,14	0,4271
Chemokine	IL-8	**0,46**	**0,0026**	0,2	0,2157	0,13	0,4499
	Eotaxin	**0,53**	**0,0004**	0,23	0,1606	0,06	0,7155
	IP-10	0,00	0,9815	−0,09	0,562	0,01	0,9483
	MCP-1	0,27	0,0832	0,16	0,327	−0,04	0,8213
	MIP-1α	**0,50**	**0,0008**	0,29	0,0709	0,08	0,6549
	MIP-1β	**0,49**	**0,001**	0,22	0,1668	−0,0003	0,9988
	RANTES	**0,42**	**0,0057**	0,25	0,1176	0,09	0,595
Inflammatory	IL-1α	0,16	0,3173	−0,02	0,9135	0,07	0,6954
	IL-1β	**0,36**	**0,0223**	0,25	0,116	0,19	0,2648
	IL-6	**0,46**	**0,0025**	0,25	0,1193	−0,06	0,7491
	IL-12p70	−0,01	0,9435	−0,21	0,1868	−0,14	0,4033
	TNF-α	**0,52**	**0,0005**	0,4	0,01	0,16	0,3509

^*^Spearman rank test used to determine correlation. 48 samples were used to evaluate the correlation of cytokine recovery between methods. Values in bold are significant, adjusted for multiple comparisons.

### Influence of blood contamination on cytokine concentrations

eCVL specimens at enrollment and visit 3 (collected third and first, respectively) were more likely to test positive for hemoglobin (3–4+) than ECS or MC (Supplementary Fig. 2A; p = 0.001 both), although this difference was not evident at visit 2. The presence of whole erythrocytes was very low at all visits and in all sample types (data not shown)^18^. With the exception of RANTES, the presence of blood (measured by hemoglobin) did not significantly influence genital cytokine concentrations in any of the sample types, at any visit, after adjusting for multiple comparisons (Supplementary Table 3). RANTES concentrations were higher in MC samples testing positive for hemoglobin (3–4+) compared to those without hemoglobin, at the enrollment visit when MC was collected first (Supplementary Table 3; Supplementary Fig. 2B). Since platelets in whole blood release RANTES^19^, it is likely that platelet-derived RANTES may account for this finding.

### Influence of sampling order on cytokine concentrations

PCA (Fig. 2) and PLSDA (Fig. 3) were used to evaluate whether the order of sample collection influenced genital cytokine profiles. Small differences were observed between each sample type (Fig. 3).

For the MC samples, the centroids for all visits were similar on X-variate 1, which explained 37% of the variation in the dataset. In contrast, the centroid for visit 1 was lower on X-variate 2 than it was for the other visits; although this explained only a relatively small proportion of the variation in the dataset (9%; Fig. 3D). Since the concentrations of most of the evaluated cytokines were inversely associated with X-variate 2 (Supplementary Table 4); the lower centroid of visit 1 samples suggests that higher cytokine concentrations in the visit 1 MC samples could be attributable to the fact that at visit 1 these samples were collected first with longer insertion periods than were used for the other sample types.

For the ECS samples, the centroid was marginally lower for visit 2 and higher for visit 3 on both X-variate 1 and 2 (Fig. 3E). In this case, the concentrations of most cytokines were inversely associated with X-variate 2 (which explained 37% of the variation in the dataset; Supplementary Table 4). This suggests that, relative to other visits, cytokine concentrations were primarily elevated in ECS samples collected at visit 2 (the visit at which ECS samples were collected first).

For the eCVL samples, the centroid for visit 1 was marginally higher on X-variate 1 and lower on X-variate 2 (Fig. 3F) than it was for the other visits. The concentrations of most of the cytokines were inversely associated with both X-variates 1 and 2 (Supplementary Table 4). Whereas this indicated that the cytokine concentrations that were associated with X-variate 1 in the visit 1 eCVL samples were lower than those found in samples taken at other visits, the cytokine concentrations associated with X-variate 2 were higher at visit 1 than they were at other visits. As X-variate 1 explained most of the variation in the dataset in this case, cytokine levels were primarily lower in eCVL samples collected at visit 1 than they were at other visits. This is possibly attributable to the visit 1 eCVL samples having been collected last.

The influence of sample collection order on cytokine concentrations was analyzed more simplistically by comparing these concentrations between ECS samples that were collected first (3-month visit) and second (enrollment) (Table 2). Some marginal differences in the absolute concentrations of RANTES, MIP-1α, IL-15 and IL-10 were found between the ECS samples collected first or second, with three of these four cytokines having lower concentations in the sample that was collected second than in the sample that was collected first. However, after correction for multiple comparisons, only IL-10 remained significantly influenced by ECS order, with a median concentration of 12.9 pg/ml (range 7.0–18.2) in ECS collected first compared to 7.8 pg/ml (range 5.2–12.1) in those collected second (Δ −5.1 pg/ml, adj. p = 0.005).

**Table 2:** Influence of ECS order on cytokine concentrations.

Cytokine functional class	Cytokine	Concentration (pg/ml) – ECS collected first (T1)			Concentration (pg/ml) - ECS collected second (T2)			Difference in medians T2-T1	P-value^*^
		Median	IQR		Median	IQR			
Inflammatory	IL-1b	148,1	29,38	434,4	195,6	46,3	472,7	47,5	0,49
	IL-1a	102,4	47,8	225,5	103,8	51,41	224,3	1,4	0,6559
	IL-6	124	36,82	332,7	86,88	29,7	176,7	−37,12	0,1354
	IL-12	18,42	10,72	28,26	13,83	5,84	29,74	−4,59	0,6474
	TNF-a	33,96	7,305	64,77	15,25	6,99	39,64	−18,71	0,0553
Chemokine	IL-8	1084	172,5	1390	619,9	271,6	1686	−464,1	0,8711
	MIP-1a	14,87	2,0	45,05	7,99	2,93	18,05	−6,88	0,0134
	MIP-1b	198,9	40,82	319,4	126,6	55,86	231,1	−72,3	0,2679
	IP-10	98,16	34,98	493,1	131,4	16,37	717	33,24	0,8881
	MCP-1	74,88	13,31	267,8	44,25	15,83	120,3	−30,63	0,0927
	RANTES	27,4	5,09	73,14	39,15	15,64	111,4	11,75	0,0216
	Eotaxin	1,68	0,06	7,905	0,06	0,06	3,27	−1,62	0,0978
Adaptive	IL-2	0,02	0,02	1,755	0,02	0,02	0,89	0	0,9579
	IL-4	0,555	0,2975	0,8675	0,47	0,23	0,84	−0,085	0,3899
	IL-5	0,325	0,01	0,62	0,27	0,01	0,55	−0,055	0,4712
	IL-13	1,48	0,6025	2,195	1,41	0,68	2,27	−0,07	0,7068
	IL-15	4,75	0,0175	8,653	2,31	0,01	5,4	−2,44	**0,016**
	IL-17	20,08	9,313	30,22	13,31	6,19	24,98	−6,77	0,5316
	IFNg	27,49	13,67	46,68	17,45	11,38	39,02	−10,04	0,1668
Growth Factors	IL-7	4,58	1,98	7,958	2,48	1,55	6,98	−2,1	0,5327
	IL-9	3,56	2,453	4,785	2,81	1,36	4,25	−0,75	0,2416
	GMCSF	56,08	34,46	67,73	59,13	41,77	73,7	3,05	0,6165
	GCSF	1523	173,2	3208	682,7	119,6	1784	−840,3	0,0971
	VEGF	310,8	154,3	515	212,2	116,1	487,8	−98,6	0,6571
	FGFb	14,87	7,29	20,78	12,91	8,23	17,35	−1,96	0,8757
	PDGF-bb	9,995	6,518	16,17	8,87	5,35	18,7	−1,125	0,6396
Anti-inflammatory	IL-10	12,86	6,955	18,23	7,83	5,21	12,1	−5,03	0,0046
	IL-1RA	13106	3623	39843	23005	4553	43411	9899	0,15

^*^Wilcoxon matched paired test used to calculate P-values.

## Discussion

There is an urgent need to standardize methodologies to collect FGT mucosal samples, especially in the context of HIV prevention, cervicovaginal cancers and reproductive outcomes. This study evaluated two of the most common sampling techniques (eCVL and ECS) for collecting genital tract samples for cytokine measurement and compared these with MC, in a longitudinal cohort study among WLHIV in which sampling method was rotated at each study visit. Comparing the concentrations of several inflammatory, adaptive, and regulatory cytokines, growth factors and chemokines, we found that cytokines were detected at higher concentrations in MC samples than either ECS or eCVL, likely because MC samples were more concentrated and offered greater ability to accurately control the dilution factor. However, cytokine concentrations generally correlated well between samples in matched sets. Importantly, sampling order only marginally influenced overall cytokine profiles captured by the three methods since greater similarity in cytokine profiles was noted between visits (where order was rotated) for each method, than between different sample types.

In contrast to the MC, for which exact volumes were determined, the comparative dilution factors for the eCVL (collected in 5 ml saline) and the ECS (swab inserted into 1.5 mL volume PBS) could not be accurately determined. Using lithium chloride to evaluate the dilution factor in CVLs, Belec *et al*.^20^ estimated a ~10-fold dilution of genital fluid for a flushed volume of 3 mL of PBS while Mitchell *et al*.^21^ estimated a ~14-fold dilution for an initial volume of 7 mL of saline flushed into the compartment. Both studies were however associated with relatively wide ranges of dilution factors for matching volumes, with some exceeding 18%. Jespers *et al*.^11^ estimated that the mean volume of undiluted fluid recovered by cervical swabbing is around 200 µL, although the exact volume was difficult to accurately determine.

We found that MC sampling from the lower genital tract of WLHIV generally resulted in significantly higher concentrations of IL-1α (produced by neutrophils, macrophages, endo- and epithelial cells)^22^, IL-12p70 (produced by dendritic cells predominantly)^23^, PDGF-bb (active during tissue healing and repair)^24^, and VEGF (responsible for vasculogenesis and angiogenesis)^25^ than ECS or eCVL. In contrast, chemokine detection was mixed, with ECS containing slightly higher concentrations of MIP-1α and MIP-1β (produced by macrophages after stimulation with bacterial endotoxins, crucial during inflammation, responsible for activating neutrophils and inducing synthesis of other pro-inflammatory cytokines such as IL-6), MCP-1 (recruits monocytes, memory T cells, and DCs to the site of inflammation produced by either tissue injury or infection) and IL-6 (secreted by T cells and macrophages)^26^. Although inflammatory cytokines were well represented in MC and ECS samples, preferential measurement of growth factors associated with tissue repair and angiogenesis in MC may reflect signatures from endometrial cells from the upper reproductive tract. Although MC and ECS are essentially sampling the same region of the lower reproductive tract, this finding could reflect the difference in duration for sample collection with the longer duration of MC sampling allowing collection of secretions more closely resembling those from the endocervical canal and possibly some from the upper reproductive tract while the transient ECS collection may only detect *in situ* cytokines that are most abundant. It is also worth noting that the overall cytokine profiles largely correlated by sampling method, with the overall ranks for each cytokine (considering their relative abundance) being conserved. In addition, no cytokine was exclusively present or completely absent by method or study visit.

However, ECS sampling appeared to be inferior to both eCVL and MC for measurement of certain chemokines like IP-10 (at all visits) and eotaxin (particularly at the visit at which ECS was collected first). IP-10 is secreted by several cell types, including endothelial cells, fibroblasts and monocytes in response to IFN-γ, which in turn acts as a chemo-attractant cytokine for cells of the monocyte/macrophage lineage, dendritic cells, NK and T cells^27^. IP-10 has also been shown to promote T cell adhesion to endothelial cells during extravasion and movement of T cells into tissue sites^28^. Because IP-10 is secreted by endothelial cells rather than epithelial cells, the inferior performance of the ECS sample compared to MC and eCVL was surprising given the very directed collection of genital secretions from the endocervical swab. Eotaxin (CCL11) is an eosinophil chemotractic protein^29^. While IP-10 concentrations across sample types were detected at median concentrations ranging from 200 pg/ml (in ECS and eCVL) to 990 pg/ml (MC) across sampling methods, eotaxin was typically detected at much lower concentrations. Since both epithelial and endothelial cells of the FGT produce cytokines, in addition to other abundant innate (including neutrophils, monocytes, macrophages, dendritic cells and NK cells) and adaptive (including T and B cells) cells resident in or recruited to this site, we are not able to determine the cellular origin of the cytokines.

Mucosal sampling is often included in clinical trials related to HIV prevention and pathogenesis. However, it is unclear whether multiple collection methods influence subsequent immune measurements so evaluating whether order of sampling matters is critical. Previously, we and others have found that mucosal sampling may cause subtle changes in the innate environment in the lower FGT, although changes were small and were only found in the context of sampling that disrupted the mucosal epithelium, such as during a Pap smear or cryoloop surgery for cervical intraepithelial neoplasia (frequently associated with visible bleeding)^30,31^. Furthermore, the kinetics of expression differ by cytokine and typically take hours rather than minutes. Schirmer *et al*.^32^ demonstrated that IL-17 production generally increased steadily over seven days while inflammatory cytokines IL-1β, IL-6 and TNF-α increased very rapidly initially, reaching a maximum within the first 24 hours. While IL-6 and TNF-α decreased rapidly thereafter, IL-1β remained elevated for the duration of the study. Although pathogen-associated molecular pattern (PAMP) molecules within the lower FGT are likely to contribute to inflammatory changes, sterile inflammation associated with damage-associated molecular pattern (DAMP) molecules, following tissue stress or injury, may contribute to amplify TLR activation^33^. It is therefore unlikely that cytokine responses from tissue trauma (from sampling) would influence cytokine profiles that typically last <3 hours.

We found only small differences between the same sample methods collected in different orders. In general, samples that were collected first tended to be associated with elevated cytokine concentrations, while cytokines collected last were associated with lower cytokine concentrations. However, few significant differences were observed when comparing the concentrations of individual cytokines measured in the same sample methods at different visits. This suggests that collection of respective samples does not result in the release of cytokines or the induction of cytokine responses that would influence subsequent samples within the timeframe of a study visit and that sequential sampling very marginally reduces the amount of available genital secretions for subsequent sample collection.

While this study aimed to evaluate whether sampling order mattered, it is possible that cytokines would not give the full story as to changes that mucosal trauma associated with sampling could induce. Although cytokines are involved in the process of tissue re-epithelialization following mucosal trauma^34^, matrix metalloproteinases (MMPs) and tissue inhibitors of metalloproteinases (secreted by macrophages and fibroblasts) likely also play a key role in mucosal wound repair and tissue remodeling, as suggested from cutaneous epithelial studies^35^.

Our study had some limitations. While we speculate that MC, ECS and eCVL collect secretions from distinct but overlapping regions of the lower FGT, we are unable to reliably distinguish the relative contribution of secretions from the endocervix, ectocervix or vagina in genital cytokine profiles. Some studies have found that cytokine production by endothelial cells is polarised with certain cytokines being preferentially secreted towards the basolateral surface of the monolayer while others are secreted apically^36^, which could have influenced some of the variation in cytokines by sampling method.

In this longitudinal study of genital cytokine profiles in WLHIV in which three collection methods were compared, rotated by study visit, to evaluate whether mucosal sampling order mattered, we conclude that MC collection was superior to ECS and eCVL, with greater recovery of cytokines, standardized dilution and lower variation between samples. We found that the order of sample collection only marginally influenced cytokine profiles. The findings from this study, that cytokine profiles are more closely clustered by sampling method than order, offer important reassurance that order probably has little influence on subsequent cytokine measurement.

## Materials and Methods

### Cohort

Eligible women were 18–40 years old, HIV-infected and had CD4 > 350 cells/mm^3^, not pregnant or wanting to become pregnant in the next two years, intending to reside in the area for the next 30 months, and without any condition contraindicating IUD use. The study was approved by ethics committees of the University of Cape Town, South Africa (reference number 283/2012) and FHI 360 (reference number 398733-61), United States, and all women provided written informed consent prior to enrollment. The authors confirm that all methods were performed in accordance with the relevant guidelines and regulations. At screening and all follow-up visits, women were tested for *Neisseria gonorrhoeae* and *Chlamydia trachomatis* via NG/CT Xpert® (Cepheid Diagnostics, Sunnyvale, California, USA), *Trichomonas vaginalis* and BV via OSOM® BV Blue and Trichomonas (Sekisui Diagnostics, Lexington, Massachusetts, USA), and *Treponema pallidum* (TP) with Alere® Determine® Syphilis (Alere Diagnostics, San Diego, California, USA) rapid tests for whole blood, and treated for reactive tests. RTI testing was performed using a dedicated vaginal swab for BV and *T. vaginalis* and ECS for *N. gonorrhoeae* and *C. trachomatis*. Women testing negative or within two weeks of treatment for any RTIs were eligible to enroll, with IUD insertion occurring at the end of the enrolment visit after all specimen collection.

### Genital tract sampling collection and processing

All genital samples were stored at 4 °C until processing, which occurred within four hours of collection.

MC: Women either self-inserted (enrolment) or had a clinician insert (3-month, 6-month) an individually wrapped single-use Instead Softcup^TM^ (Evofem Inc., San Diego, CA, USA). Self-insertion was only feasible at the enrolment visit, based on the order of specimen collection. The MC was inserted to cover the cervix and remained in place for one hour before being removed by a clinician and transferred to a sterile 50 mL Falcon tube. Clinicians recorded the exact time of MC insertion and removal. At the laboratory, the tube containing the MC sample was weighed and the volume of secreted fluid calculated (used MC-mean weight of ten 50 mL tubes containing an unused MC), as previously described^18^. The MC membrane was inverted using a sterile wooden disposable spatula, after which the tube was centrifuged at 1500 rpm for 10 minutes to allow the content to collect at the bottom. Residual secretions on the MC were removed by pipette after centrifugation, the MC removed and discarded. Assuming 1 g = 1 mL, MC secretions were diluted 10 times with PBS.

ECS: A clinician collected endocervical fluid with an elongated tapered flocked swab (FLOQ swab, Copan Diagnostics, Murrieta, CA, USA) during the pelvic examination. Each swab was allowed to absorb fluid inside the endocervical canal for 60 seconds, without rotating the swab to prevent micro-abrasion. Swabs were placed into cryovials containing 1.5 mL PBS, for transport to the laboratory. In the laboratory, ECS samples were vortexed for 1 minute to suspend and mix secretions in PBS. All fluid was removed from the swab by gently scraping the swab head against the inside of the tube before discarding it. ECS secretions were aliquoted and stored at −80 ^o^C for cytokine measurement.

eCVL: Previous studies have suggested that swab-enriched CVLs are superior to standard CVLs for measurement of genital tract HIV concentrations in WLHIV^8,9^. For eCVL collection, an elongated tapered flocked swab was first used to sample from the cervix as described for the ECS, and then inserted into a transport tube. This was followed by a cervical lavage with 5 mL normal saline introduced using a flexible plastic pipette into the posterior vaginal fornix for 60-seconds (with three consecutive aspirations and discharges against the vaginal walls and the cervix). Total lavage fluid was recovered from the posterior fornix by a final aspiration and added to the transport tube with the cervical swab. At the laboratory, eCVL specimens were centrifuged at 1500 rpm for 10 minutes with the swabs in the tube. The supernatant was aspirated, aliquoted and stored at −80 ^o^C for cytokine measurement.

While PBS was used for dilution of MC and ECS specimens, normal saline was used for eCVL according to the method published previously^18^. Dezzutti *et al*.^9^ found that diluent did not impact recovery of most chemokines. The amount of diluent used for ECS and eCVL specimens was determined from our previous study^18^.

### Determining blood contamination

Prior to freezing, all genital samples were tested for hemoglobin and erythrocyte contamination using Combur^3^ Test E strips (Cobas, Roche Diagnostics Ltd., Rotkreuz, Switzerland)^18^. Hemaglobin was used as a measure of blood contamination, using a 4 point scoring system.

### Measurement of genital tract cytokines by Luminex

Concentration of 27 cytokines were measure using a Bio-Plex Pro Human Cytokine 27-plex Assay (Bio-Rad Laboratories Inc., USA), while IL-1a was measured alone using a single bead array. Cytokine plates were read using a Bio-Plex Suspension Array Reader (Bio-Rad Laboratories Inc., USA). Data were analyzed using Bio-Plex manager software (version 4). Cytokine levels that were below the lower limit of detection of the assay were reported as the mid-point between zero and the lowest concentration measured for that given cytokine. As samples were spread across twenty plates and run over a period of three weeks, a reference panel of nine MC samples was included on each of the plates (inter-plate controls), in addition to three matching sets of MC, ECS, and eCVL being duplicated on each set of plates (intra-plate controls). Spearman’s rank to measure intra-assay and inter-assay correlation coefficients was used to determine assay reliability and reproducibility (Supplementary Table 2). Within-plate reproducibility was evaluated using Spearman’s rank correlation coefficients between the duplicate samples on the 10 plates. Intraplate correlation coefficients ranged from 0.66–0.97, with four cytokines having correlation coefficients lower than 0.80 (IL-2, IL-15, GM-CSF and eotaxin; Supplementary Table 2). Inter-plate reproducibility calculated as the overall median of all interplate correlation coefficients ranged between 0.57 to 1, with the same four cytokines having a coefficient lower than 0.80. The variation in measured cytokine concentrations was highest in the most poorly detected cytokines as is reflected by IL-2 (46.9%), eotaxin (59.4%) and IL-15 (67.4%), all having intra-plate and inter-plate correlation coefficients <0.80.

### Statistical Analysis

Paired comparisons between collection methods were analyzed with Wilcoxon rank sum tests. Associations between the levels of cytokine measured by the three methods were examined using Spearman rank correlation. For the MC, we also estimated Spearman’s rank correlation between each cytokine and insertion time and weight of MC specimen respectively. Factor analysis was used to reduce the complexity of the dataset, as we have previously described^4^, and cytokines in each functional group (pro-inflammatory, chemokine, hematopoietic, growth factor, adaptive and regulatory) were loaded onto a single factor to generate the estimates of each group. Confirmatory factor analysis was used to group cytokines according to their primary biological functions and generate factor scores for each functional group that were then used for downstream analyses. Logistic regressions were carried out to assess the impact of sampling techniques on genital cytokine levels and factor scores: β-coefficients generated from the models indicate the degree of association between each sampling technique and factor scores or cytokine concentrations. Unsupervised hierarchical clustering, PCA and PLSDA analyses were performed in R using the package mixOmics^37^. Cross-validation errors for PLSDA were generated by partitioning the dataset into ten subsets, training the model on nine of these subsets and testing it on one, which was then repeated 50-times for each model^37^. STATA™ version13 (StataCorp,USA) and R were used for analyses.

### Data availability statement

All datasets generated during and/or analysed during the current study are available from the corresponding author on request.

## Electronic supplementary material

Supplementary Figures and Tables
