# Supplementary material for: Lower genital tract cytokine profiles in South African women living with HIV: influence of mucosal sampling
Source: Sci Rep. 2018 Aug 15;8:12203. doi: 10.1038/s41598-018-30663-8 (PMC6093917; doi:10.1038/s41598-018-30663-8)
Supplement: Supplementary file 1 — Supplementary Figures and Tables [file 41598_2018_30663_MOESM1_ESM.pdf]

Lower genital tract cytokine profiles in South African women living with HIV: influence of mucosal sampling

Shameem Z. Jaumdally, PhD,[1&] Lindi Masson, PhD,[1,4] Heidi E. Jones, PhD, MPH,[2] Smritee Dabee, MSc,[1] Donald R. Hoover, PhD, MPH,[3] Hoyam Gamieldien, MSc,[1] Nontokozo Langwenya, MPH,[5] Landon Myer, MD, PhD,[5] Catherine S. Todd, MD, MPH[6#], Jo-Ann S. Passmore, PhD[1,4,7,8#]*

Supplementary Figure 1. Comparison between (A) inflammatory cytokine, (B) chemokine, (C) adaptive cytokines, (D) growth factors, and (E) anti-inflammatory concentrations by sampling method (MC, ECS and eCVL) and sampling order. For each figure set: (i) Each data point represents an individual participant's cytokine concentration in one sample (MC-left panel; ECS-middle; eCVL-right panel) over three time points (0, 3, and 6 months), with sampling order indicated in brackets. Solid lines are used to connect participant cytokine levels over time. (ii) Box-and-whisker plots showing enrollment cytokine concentrations in the three distinct sample types. The box shows median, 25th and 75th percentile; while whiskers show the 5th and 95th percentiles. * indicates $p<0.05$ compared to MC, while ** indicates $p<0.01$ (Wilcoxon matched-pairs test).

Supplementary Figure 2. Hemaglobin contamination of genital samples influence RANTES concentrations detected. (A) Hb levels (0-4+) according to sampling method at visit 1 (v1) and 3 (v3); Fishers exact test was used to compare Hemaglobin contamination (3+) in ECS or eCVL to MC, and p-values <0.05 were considered significant. (B) RANTES concentrations detected in MC samples from visit 1 and 3, by hemaglobin grade (0 Hb – clear boxes; 1 Hb – yellow boxes; 2 Hb – peach boxes; 3 Hb – orange boxes; 4 Hb – red boxes). Mann-whitney U test was used to compare RANTES concentrations in 1-4 group compared to 0 Hb group and p-values <0.05 were considered significant.

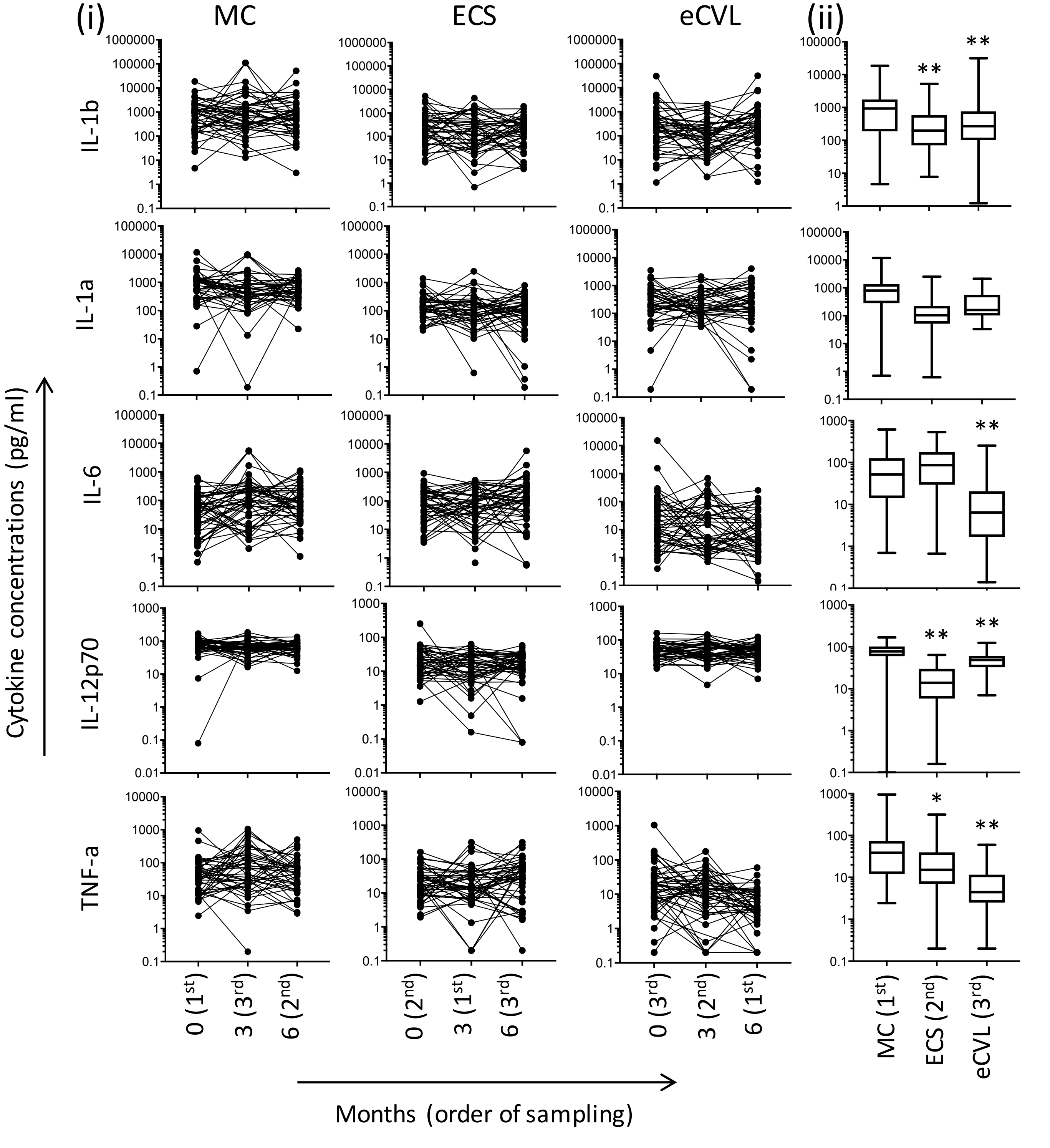

Supplementary Figure 1A
Inflammatory cytokines

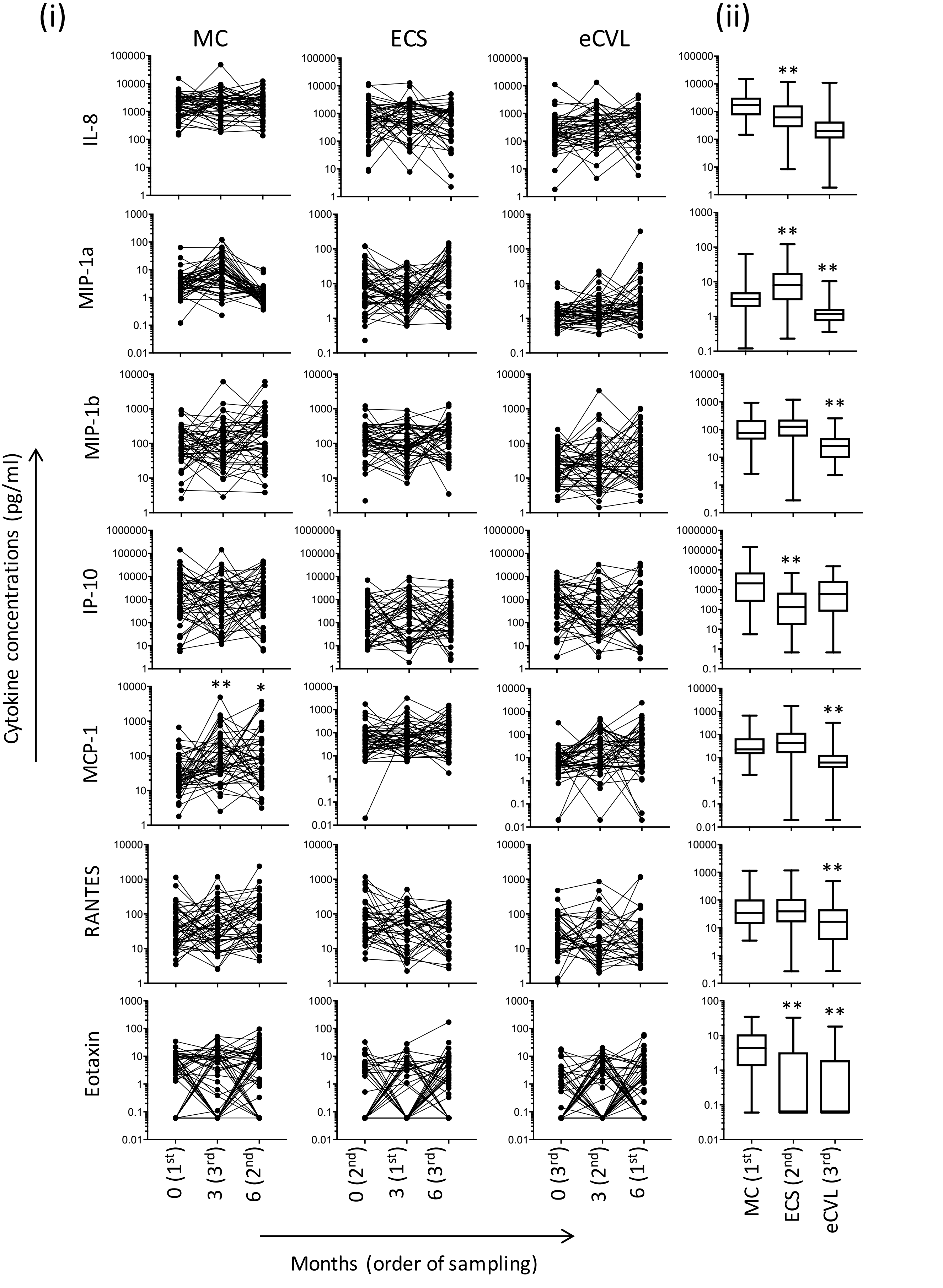

Supplementary Figure 1B
Chemokines

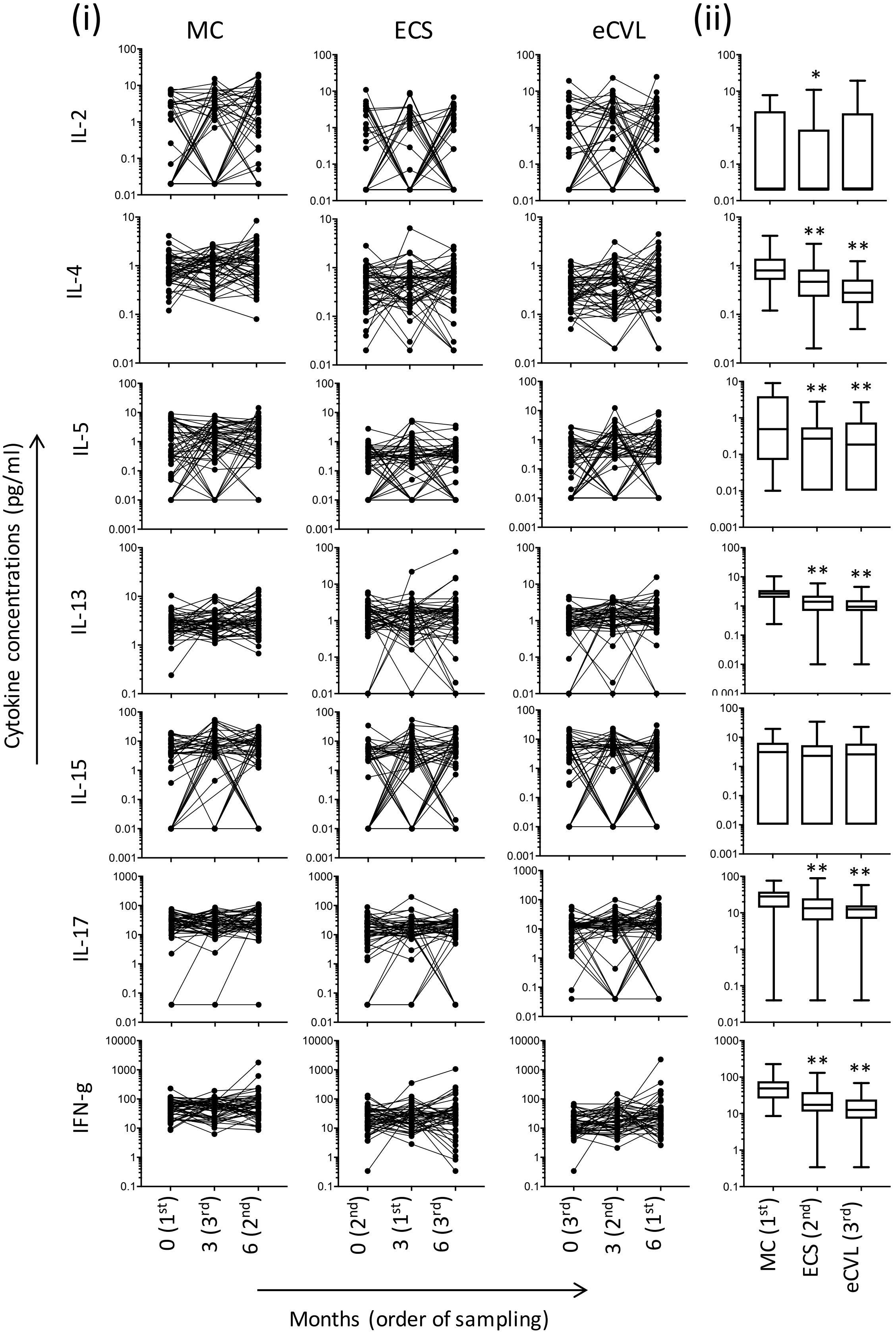

Supplementary Figure 1C
Adaptive Cytokines

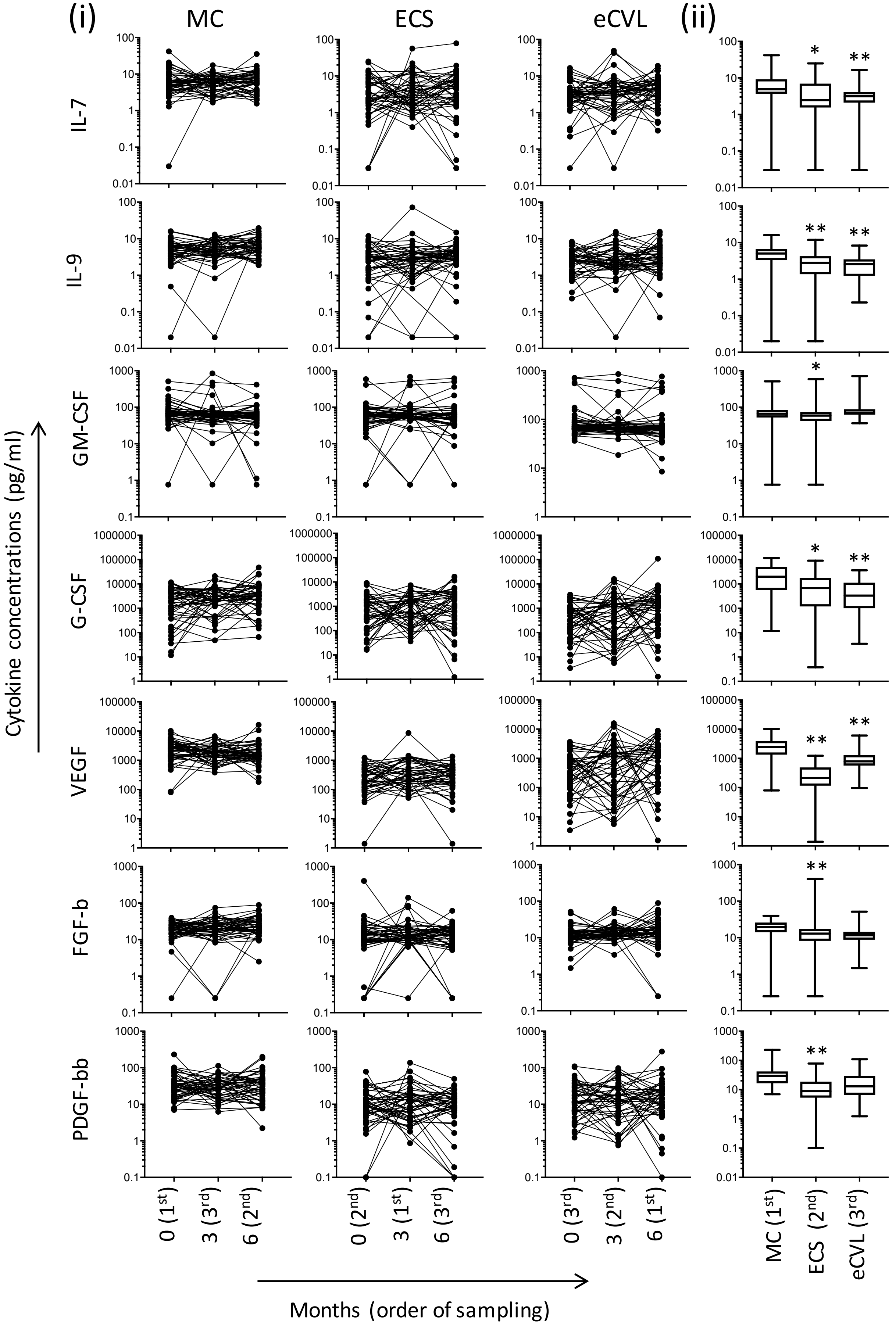

Supplementary Figure 1D
Growth Factors

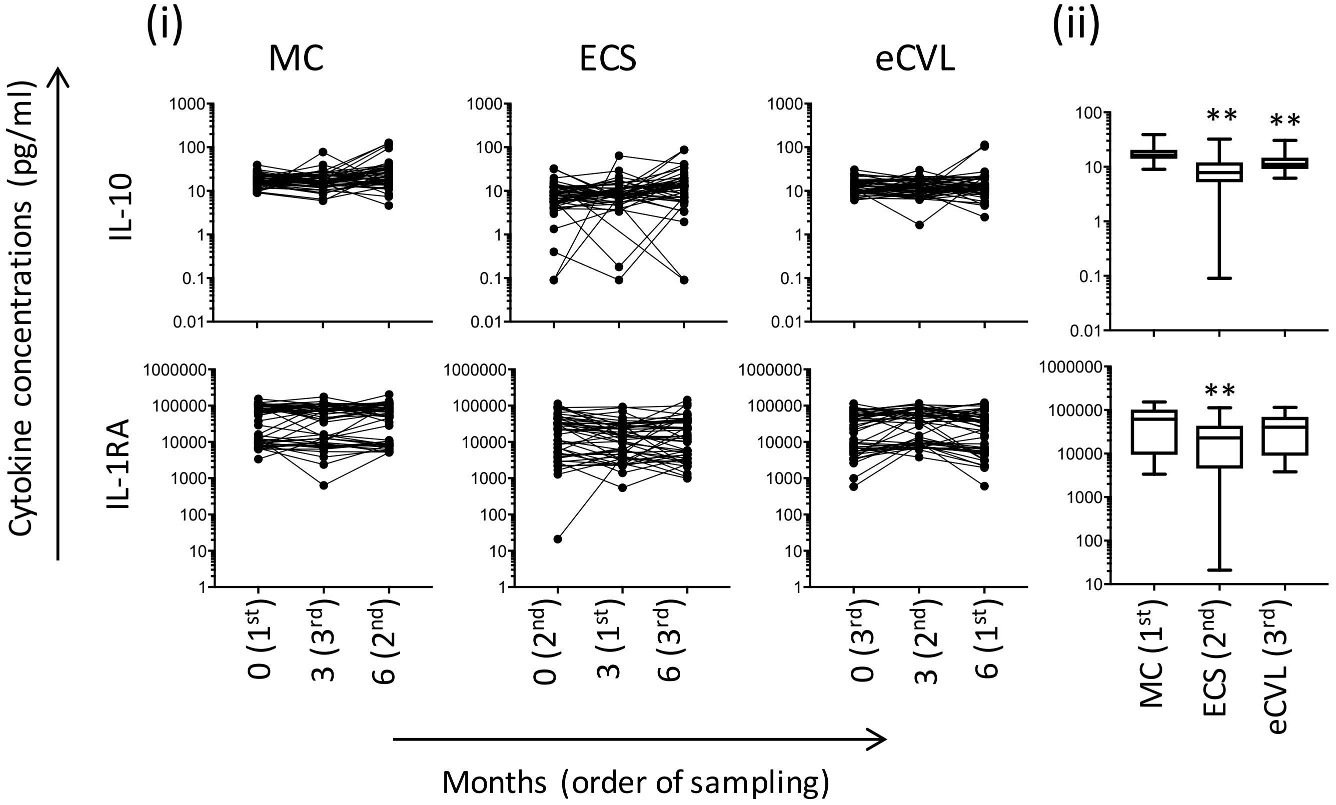

Supplementary Figure 1E
Regulatory

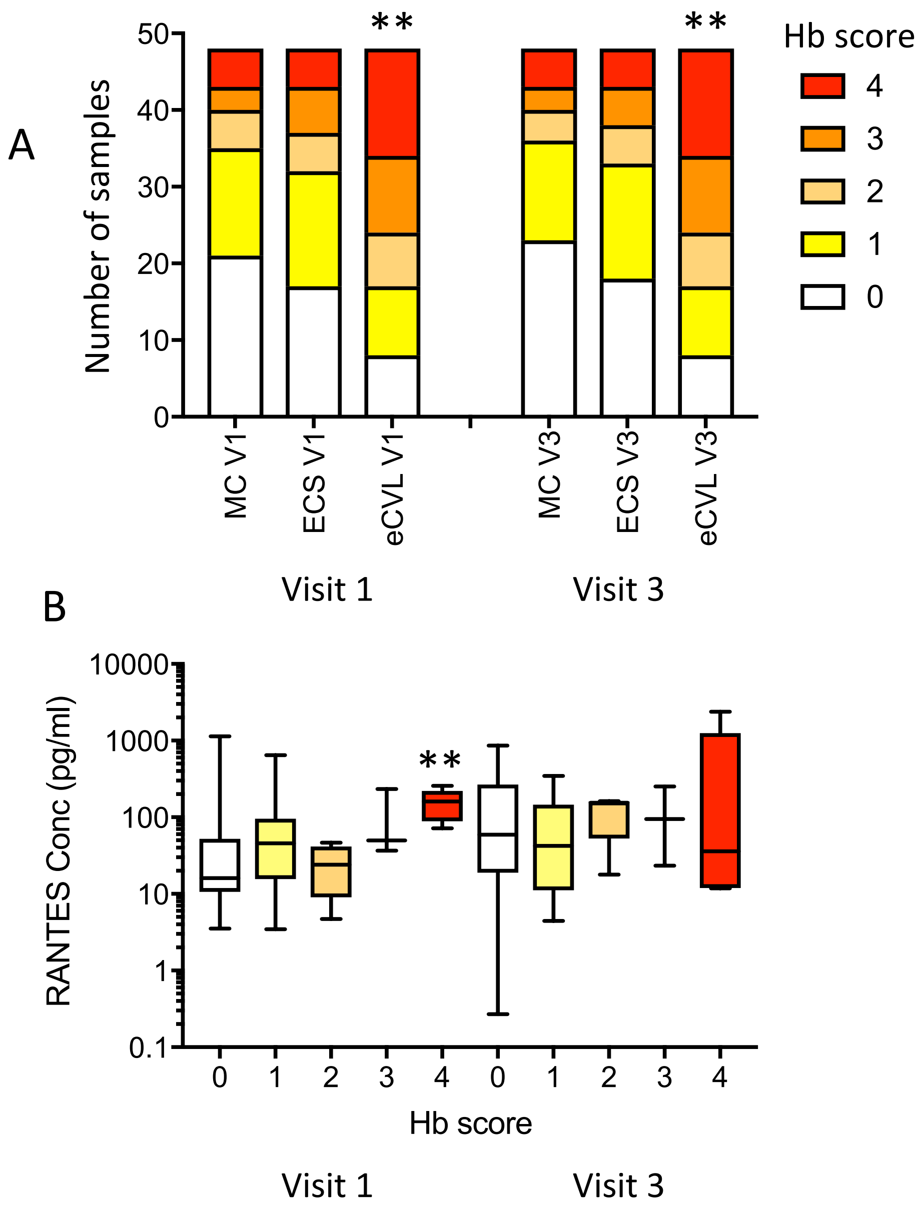

A
Number of samples
50
40
30
20
10
0
**
**
Hb score
4
3
2
1
0
MC V1
ECS V1
eCVL V1
MC V3
ECS V3
eCVL V3
Visit 1
Visit 3
B
RANTES Conc (pg/ml)
10000
1000
100
10
1
0.1
**
0
1
2
3
4
0
1
2
3
4
Hb score
Visit 1
Visit 3

Supplementary Table 1: Correlation* between cytokine concentrations in matched sample sets (baseline)

Class	Cytokine	MC vs ECS		MC vs eCVL		ECS vs eCVL	
		Rho	P-value	Rho	P-value	Rho	P-value
Regulatory	IL-1ra	0.79	<0.0001	0.81	<0.0001	0.80	<0.0001
	IL-10	0.32	0.0251	0.46	0.0009	0.44	0.0016
Adaptive	IL-2	0.66	<0.0001	0.64	<0.0001	0.75	<0.0001
	IL-4	0.16	0.2903	0.32	0.0290	0.19	0.2061
	IL-5	0.35	0.0164	0.62	<0.0001	0.44	0.0019
	IL-13	0.04	0.7935	0.39	0.0063	0.20	0.1766
	IL-15	0.71	<0.0001	0.70	<0.0001	0.64	<0.0001
	IL-17	0.47	0.0008	0.61	<0.0001	0.53	0.0001
	IFN-γ	0.10	0.5179	0.42	0.0033	0.22	0.1276
Growth Factors	IL-7	0.18	0.2149	0.33	0.0221	0.55	<0.0001
	IL-9	0.48	0.0005	0.50	0.0003	0.68	<0.0001
	FGF basic	0.55	<0.0001	0.52	0.0001	0.50	0.0003
	G-CSF	0.34	0.0010	0.44	0.0020	0.50	0.0003
	GM-CSF	0.19	0.1949	0.11	0.4568	0.63	<0.0001
	PDGF-bb	0.07	0.6505	0.42	0.0032	0.28	0.0569
	VEGF	0.24	0.1002	0.47	0.0007	0.41	0.0039
Chemokine	IL-8	0.20	0.1671	0.28	0.0559	0.23	0.1130
	Eotaxin	0.40	0.0052	0.55	<0.0001	0.50	0.0003
	IP-10	0.32	0.0252	0.74	<0.0001	0.56	<0.0001
	MCP-1	0.30	0.0384	0.45	0.0012	0.42	0.0028
	MIP-1α	0.18	0.2262	0.32	0.0284	0.10	0.4950
	MIP-1β	0.43	0.0021	0.64	<0.0001	0.48	0.0006
	RANTES	0.17	0.2446	0.39	0.0068	0.58	<0.0001
Inflammatory	IL-1α	0.29	0.0553	0.44	0.0027	0.48	0.0010
	IL-1β	0.42	0.0027	0.64	<0.0001	0.48	0.1000
	IL-6	0.42	0.0027	0.34	0.0187	0.27	0.0634
	IL-12p70	0.18	0.2311	0.38	0.0082	0.47	0.0008
	TNF-α	0.28	0.0509	0.47	0.0008	0.38	0.0085

*Spearman rank test used to determine correlation. 48 samples were used to evaluate the correlation of cytokine recovery between methods.

Supplementary Table 2: Reproducibility* of cytokine measurement by methodology

Class	Cytokine	MC		ECS		eCVL	
		Rho	p-value	Rho	p-value	Rho	p-value
Regulatory	IL-1ra	0.95	<0.0001	0.86	<0.0001	0.93	<0.0001
	IL-10	0.93	<0.0001	0.94	<0.0001	0.84	<0.0001
Adaptive	IL-2	0.68	<0.0001	0.61	0.0003	0.59	0.0007
	IL-4	0.92	<0.0001	0.75	<0.0001	0.88	<0.0001
	IL-5	0.96	<0.0001	0.82	<0.0001	0.96	<0.0001
	IL-13	0.85	<0.0001	0.77	<0.0001	0.91	<0.0001
	IL-15	0.82	<0.0001	0.62	0.0002	0.58	0.0007
	IL-17	0.81	<0.0001	0.83	<0.0001	0.61	0.0004
	IFN-γ	0.91	<0.0001	0.84	<0.0001	0.92	<0.0001
Growth Factors	IL-7	0.90	<0.0001	0.75	<0.0001	0.86	<0.0001
	IL-9	0.97	<0.0001	0.59	0.0006	0.78	<0.0001
	FGFb	0.86	<0.0001	0.80	<0.0001	0.74	<0.0001
	G-CSF	0.98	<0.0001	0.82	<0.0001	0.95	<0.0001
	GM-CSF	0.85	<0.0001	0.73	<0.0001	0.82	<0.0001
	PDGF-bb	0.90	<0.0001	0.74	<0.0001	0.86	<0.0001
	VEGF	0.94	<0.0001	0.94	<0.0001	0.95	<0.0001
Chemokine	IL-8	0.94	<0.0001	0.95	<0.0001	0.95	<0.0001
	Eotaxin	0.72	<0.0001	0.96	<0.0001	0.61	0.0004
	IP-10	1.00	<0.0001	0.81	<0.0001	0.99	<0.0001
	MCP-1	0.93	<0.0001	0.81	<0.0001	0.97	<0.0001
	MIP-1α	0.94	<0.0001	0.94	<0.0001	0.89	<0.0001
	MIP-1β	0.96	<0.0001	0.93	<0.0001	0.96	<0.0001
	RANTES	0.94	<0.0001	0.86	<0.0001	0.95	<0.0001
Inflammatory	IL-1α	0.78	<0.0001	0.67	0.0001	0.79	<0.0001
	IL-1β	0.95	<0.0001	0.98	<0.0001	0.97	<0.0001
	IL-6	0.98	<0.0001	0.84	<0.0001	0.95	<0.0001
	IL-12p70	0.92	<0.0001	0.88	<0.0001	0.94	<0.0001
	TNF-α	0.97	<0.0001	0.91	<0.0001	0.91	<0.0001

*Reproducibility of cytokine measurements calculated by correlation between individual measurements of 9 samples (3 MC, 3 ECS and 3 eCVL) duplicated on each of the ten luminex plates used for this study. Different samples were replicated across the 10 plates, providing 30 duplicates for each method.

Supplementary Table 3. Influence of blood contamination (Hb) on cytokine levels in MC at enrollment

Cytokine functional class	Cytokine concentration (pg/ml) [median (IQR)]				Unadj. P-value#
	[Hb]	0	1-2+	3-4+	
Inflammatory	IL-1b	672,6 (137,7-1368)	941,3 (249,4-1407)	1412 (418,1-2218)	0.3645
	IL-1a	1252 (573,7-1860)	657,3 (227,4-1364)	412,7 (208,2-881,2)	0,0254
	IL-6	52,4 (13,1-106)	47,8 (15,0-146,7)	82,8 (5,0-133,7)	0.9610
	IL-12P70	78,1 (55,8-98,1)	76,7 (59,5-112,5)	80,4 (60,8-93,5)	0,8344
	TNF-a	40,8 (11,1-83)	31,7 (11,2-56,4)	44,6 (14,0-121,3)	0,6314
Chemokine	IL-8	1442 (577,8-3215)	1716 (1142-2927)	1788 (812,1-3299)	0,8070
	MIP-1a	3,2 (1,5-4,8)	2,7 (1,9-5,3)	3,7 (2,1-5,1)	0,7600
	MIP-1b	73,4 (39,7-227,8)	73,4 (16,6-221,1)	77,9 (53,9-234,5)	0,8307
	IP-10	420,4 (194,5-2350)	3974 (754,3-8568)	10320 (258-41174)	0,0568
	MCP-1	19,8 (12,5-61,8)	20,4 (11,1-30,5)	58,5 (19,0-103,3)	0,2175
	RANTES	16,1 (10,6-52,1)	36,0 (14,3-57,5)	133,1 (55,3-222,1)*	0,0069*
	Eotaxin	3,5 (0,7-10,2)	2,7 (0,06-7,6)	10,2 (5,7-12,0)	0,0502
Adaptive	IL-2	0 (0-2,7)	0,2 (0,2-2,8)	1,6 (0,02-5,2)	0,4219
	IL-4	0,8 (0,5-1,6)	0,9 (0,5-1,2)	0,8 (0,6-1,4)	0,9267
	IL-5	2,2 (0,4-5,7)	0,3 (0,05-1,3)	0,3 (0,07-4,6)	0,0611
	IL-13	2,8 (1,9-3,7)	2,8 (1,9-3,6)	1,9 (1,5-3,5)	0,4058
	IL-15	3,5 (0,01-6,1)	0,1 (0,1-4,3)	7,1 (0,3-14,4)	0,2393
	IL-17	22,2 (13,6-41,1)	16,2 (9,4-31,4)	35,7 (31,4-53,6)	0,0288
	IFN-g	50,1 (19,6-76,7)	46,6 (30,6-77,5)	58,9 (32,4-89,0)	0,7003
Haematopeotic	IL-7	4,9 (3,7-10,9)	4,9 (3,1-6,7)	5,3 (3,8-7,3)	0,8535
	IL-9	5,5 (3,9-6,7)	3,5 (2,3-7,1)	4,9 (3,5-6,4)	0,3603
	GM-CSF	68,4 (52,1-92,5)	58,9 (51,0-74,9)	66,2 (47,3-85,8)	0,7563
	G-CSF	1841 (468,1-3957)	1233 (492,4-5678)	3172 (622,3-4900)	0,8877
	VEGF	2384 (1336-3343)	2462 (1135-5025)	2333 (1355-3497)	0,7056
	FGF-b	19,7 (12,1-28,1)	17,3 (14,2-21,2)	25,7 (16,2-32,6)	0,1891
	PDGF-bb	21,0 (12,9-34,2)	34,5 (23,5-41,4)	46,2 (29,2-87,1)	0,0319
Anti-inflammatory	IL-10	17,9 (14,9-21,7)	16,3 (11,7-20,6)	15,7 (14,6-17,8)	0,4535
	IL-1RA	92803 (19908-114468)	12267 (7643-73180)	27039 (9765-81690)	0,0255

#Kruskal-Wallis test; p-values <0.05 considered significant, adjusted for multiple comparisons using FDR stepdown procedure.

Supplementary Table 4: Partial Least Squares Discriminant Analysis (PLSDA) cytokine loadings

Class	Cytokine	MC		ECS		eCVL	
		X-variate 1	X-variate 2	X-variate 1	X-variate 2	X-variate 1	X-variate 2
Pro-inflammatory	IL-1α	0.115	-0.133	-0.273	-0.032	0.068	-0.221
	IL-1β	-0.075	-0.283	-0.125	-0.203	-0.302	-0.006
	IL-6	-0.270	0.018	0.218	-0.224	-0.255	-0.088
	IL-12p70	0.046	-0.122	-0.183	-0.229	-0.110	-0.040
	TNF-α	-0.173	-0.234	0.226	-0.187	-0.371	0.055
Chemokine	Eotaxin	-0.096	-0.304	0.382	-0.114	-0.244	0.121
	IL-8	-0.099	-0.234	-0.173	-0.268	-0.152	-0.210
	IP-10	0.014	-0.255	-0.098	-0.166	0.119	-0.438
	MCP-1	-0.344	0.283	0.191	-0.248	-0.280	-0.016
	MIP-1α	-0.368	0.065	0.437	-0.072	-0.300	-0.021
	MIP-1β	-0.203	-0.162	0.236	-0.180	-0.184	-0.118
	RANTES	-0.151	-0.187	-0.234	-0.177	0.095	-0.443
Growth factors	IL-7	-0.115	-0.188	0.037	-0.224	-0.169	-0.146
	IL-9	-0.205	-0.045	0.091	-0.187	-0.070	-0.140
	FGF-b	-0.166	-0.165	-0.172	-0.252	-0.032	-0.222
	G-CSF	-0.270	0.031	0.030	-0.231	-0.216	-0.131
	GM-CSF	0.113	-0.171	-0.070	-0.069	0.083	-0.100
	PDGF-bb	0.022	-0.296	-0.217	-0.286	0.025	-0.416
	VEGF	0.127	-0.198	-0.187	-0.244	-0.188	0.051
Adaptive	IFN-γ	-0.164	-0.234	0.019	-0.211	-0.298	-0.057
	IL-2	-0.259	0.132	0.067	-0.090	-0.088	0.043
	IL-4	-0.158	-0.234	-0.012	-0.229	-0.168	-0.213
	IL-5	-0.208	0.172	-0.067	-0.091	-0.212	0.033
	IL-13	-0.150	-0.073	-0.002	-0.177	-0.219	-0.043
	IL-15	-0.268	0.272	0.180	-0.157	-0.022	0.004
	IL-17	-0.160	-0.114	-0.043	-0.220	-0.046	-0.115
Regulatory	IL-10	-0.267	-0.058	0.289	-0.099	-0.066	-0.230
	IL-1ra	-0.025	0.000	-0.002	-0.035	0.182	-0.253
